# Systematic studies into uniform synthetic protein nanoparticles

**DOI:** 10.3762/bjnano.13.22

**Published:** 2022-02-28

**Authors:** Nahal Habibi, Ava Mauser, Jeffery E Raymond, Joerg Lahann

**Affiliations:** 1 Biointerfaces Institute, University of Michigan, Ann Arbor, MI, 48109, USA; 2 Department of Chemical Engineering, University of Michigan, Ann Arbor, MI, 48109, USA; 3 Department of Biomedical Engineering, University of Michigan, Ann Arbor, MI, 48109, USA

**Keywords:** nanogels, nanomedicine, particle characterization, protein-based biomaterials

## Abstract

Nanoparticles are frequently pursued as drug delivery carriers due to their potential to alter the pharmacological profiles of drugs, but their broader utility in nanomedicine hinges upon exquisite control of critical nanoparticle properties, such as shape, size, or monodispersity. Electrohydrodynamic (EHD) jetting is a probate method to formulate synthetic protein nanoparticles (SPNPs), but a systematic understanding of the influence of crucial processing parameters, such as protein composition, on nanoparticle morphologies is still missing. Here, we address this knowledge gap by evaluating formulation trends in SPNPs prepared by EHD jetting based on a series of carrier proteins and protein blends (hemoglobin, transferrin, mucin, or insulin). In general, blended SPNPs presented uniform populations with minimum diameters between 43 and 65 nm. Size distributions of as-jetted SPNPs approached monodispersity as indicated by polydispersity indices (PDI_SEM_) ranging from 0.11–0.19. Geometric factor analysis revealed high circularities (0.82–0.90), low anisotropy (<1.45) and excellent roundness (0.76–0.89) for all SPNPs prepared via EHD jetting. Tentatively, blended SPNPs displayed higher circularity and lower anisotropy, as compared to single-protein SPNPs. Secondary statistical analysis indicated that blended SPNPs generally present combined features of their constituents, with some properties driven by the dominant protein constituent. Our study suggests SPNPs made from blended proteins can serve as a promising drug delivery carrier owing to the ease of production, the composition versatility, and the control over their size, shape and dispersity.

## Introduction

As nanoparticle platforms for drug delivery transition from novelties to foundational biomedical technologies [1–3], it is critical to augment the existing strategies with precisely engineered nanocarriers that are better equipped to maneuver the host of barriers that exist in clinical translation [4–5]. Nanoparticles made of proteins [6] hold significant promise in this respect and different methods have been adopted to fabricate protein-based nanoparticles including nab technology [1], desolvation methods [2], and self-assembly [3].

The protein human albumin is a natural carrier of endogenous hydrophobic molecules [7] and has been shown to be an attractive vehicle for hydrophobic drugs in several clinical and preclinical applications [8]. This feature is highlighted in the success of Abraxane^TM^, a nanoparticle albumin bound (nab) technology to delivery paclitaxel – a hydrophobic cancer therapeutic. Paclitaxel was traditionally formulated through solubilization with harsh solvents, which led to adverse side effects and special administration instrumentation [9]. While Abraxane^TM^ was first FDA-approved for the treatment of metastatic breast cancer, it has since been investigated for other cancers, particularly as a combination therapy [10]. To leverage the endogenous properties of albumin, nab technology uses a high-pressure manufacturing process to force hydrophobic drugs into the internal hydrophobic pockets of human serum albumin (HSA) [11]. This leads to the formation of albumin-bound, paclitaxel-loaded HSA particles with diameters of approximately 130 nm [10–11]. Since then, Abraxane^TM^ has been used for non-small cell lung cancer, late-stage pancreatic cancer, and as a treatment for metastatic breast cancer [12–13]. Nevertheless, nab-based nanoparticles suffer from significant drawbacks, such as poorly defined physical properties and/or stability in the bloodstream [14].

Desolvation methods have been used to prepare various therapeutic protein nanoparticles [15–17]. The desolvation process requires the addition of desolvating agents, such as ethanol or acetone, to induce changes in protein structure (sometimes fully denaturing the protein) and to cause subsequent precipitation of protein aggregates [18]. Self-assembly strategies also provide access to a variety of structurally diverse [19] protein nanoparticles. With the advent of in silico design and subsequent production of de novo protein nanoparticle systems, the number of specific protein building blocks that can be designed for self-assembly strategies has increased in recent years. If a protein system can successfully be designed, these synthetic proteins allow for tunable functionality and/or stability profiles [19].

Electrohydrodynamic (EHD) co-jetting is a versatile technology that has been utilized to fabricate compartmentalized microparticles and nanoparticles [20–22]. Key properties of these drug delivery carriers include tunable payload delivery kinetics, the presence of multiple drug-loaded compartments, and compatibility with more than one administration route [23–24]. More recently, reactive EHD jetting has been used for the preparation of synthetic protein nanoparticles (SPNPs) [25]. SPNPs have been used for delivery of RNAi-based therapeutics and have resulted in tumor regression and long-term survival in mice with glioblastoma multiforme [26]. Compared to methods mentioned above, EHD jetting also allows for the fabrication of multicompartmental protein particles [25]. Given the abundance of proteins and their importance in maintaining important biological functions, such as homeostasis, SPNPs based on proteins with specific biological functions, such as transferrin, insulin, albumin, mucin, or hemoglobin, may represent powerful candidates as next-generation biologics. The EHD jetting process is influenced by a number of governing principles, such as viscosity and dielectric constant of the premixture, the bias between the jetting needle and collection substrate. A stable jet is achieved with the assistance of a constant pumping rate, to generate a Taylor cone that resolves into individual microdroplets that contain a very limited amount of macromolecules. During travel from the onset of the cone jet to the substrate, the solvent system evaporates, and the resulting particles are deposited on the substrate.

To date, SPNPs have displayed a broad spectrum of sizes, swelling factors, elasticities, and mesh sizes [27]. Here, we improve on these recent efforts by elucidating the role of blended matrix proteins on the physical properties of the respective nanoparticles. Specifically, this work systematically explores the relationship between SPNP formulation parameters and nanoparticle morphology, while also providing detailed insights into size distributions and uniformities.

## Results and Discussion

### Single-protein SPNPs

**Figure 1:**
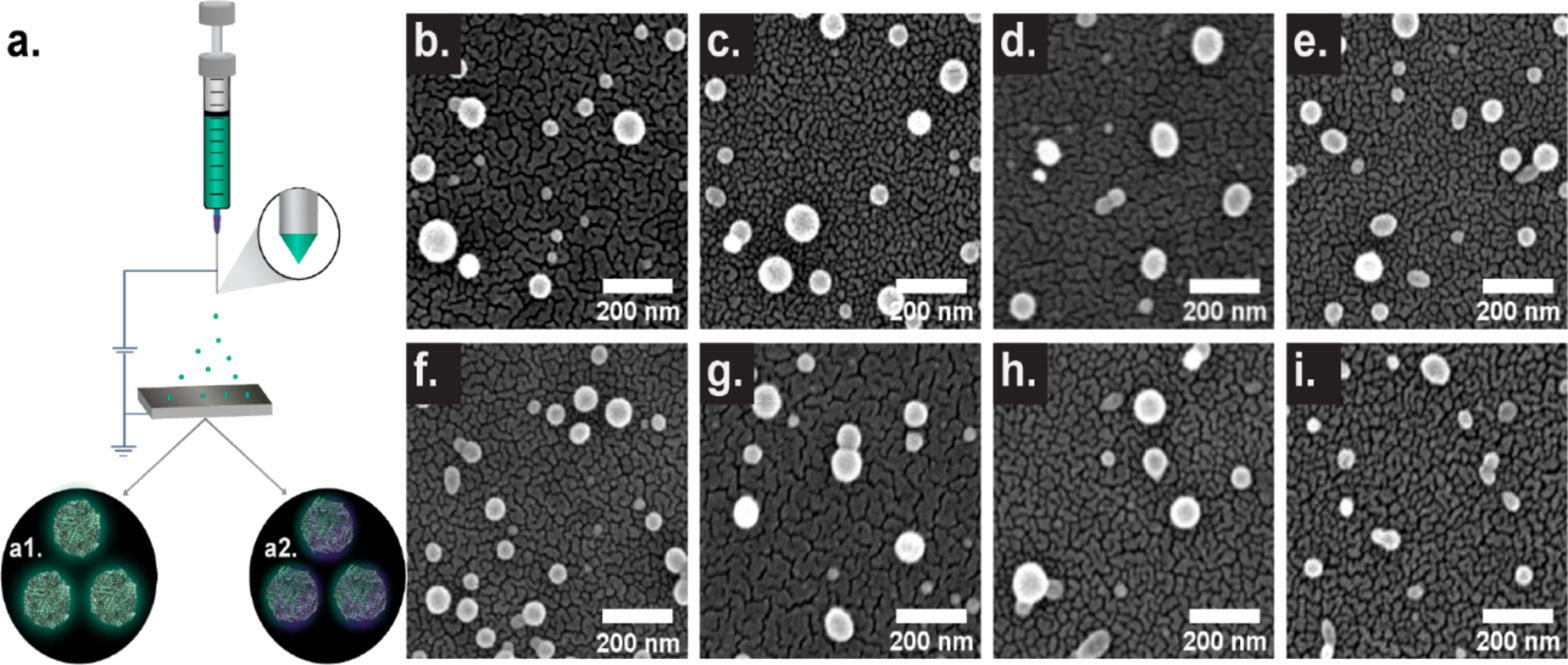
EHD jetting process and SEM images of characteristic SPNPs. (a) Synthesis of various SPNPs comprised of (a1) single proteins or (a2) binary protein blends, and PEG macromers. (b–i) SEM images of single protein and blended SPNPs. (b) HEM/HSA, (c) TF/HSA, (d) MUC/HSA, (e) INS/HSA, (f) HEM, (g) TF, (h) MUC, (i) INS. Scale bar: 200 nm.

A range of SPNP formulations were prepared via EHD jetting from hemoglobin (HEM), transferrin (TF), mucin (MUC), insulin (INS), and human serum albumin (HSA) (Figure 1a).

Generally, dilute solutions of a protein at 10% (w/v) in a 9:1 (v/v) mixture of water and ethanol were used for all jetting experiments. This inclusion of ethanol decreased the dielectric constant and surface tension of the solution. The exception was insulin- and mucin-based SPNPs, which were manufactured as described in the Materials section. Furthermore, a homobifunctional amine-reactive macromer, NHS-PEG-NHS, was added to the jetting solution at 10% w/w_protein_. Application of a bias (voltage) between the needle and collection tray resulted in a field that distorts the solution meniscus into a Taylor cone. The charged solution was accelerated downward to form droplets. Rapid solvent evaporation occurred during jetting, with solid SPNPs deposited on the collection plate. Completion of the crosslinking was achieved through SPNP storage at 37 °C for 7 days.

Multiple studies have shown that nanoparticle formulation, size, and surface chemistry are not the only factors governing the biological response at the cellular level. Several studies [28–30] have shown that considerations such as aspect ratio, stiffness/deformability, and particle surface roughness (deviation of circularity) can have a comparable impact on cellular uptake and/or endosomal escape. Hence, it is important to incorporate thorough shape analysis into the assessment of emerging platforms from the very onset of their expansion into the general nano-bio research. The solution-based corollary to dry-state imaging that can provide the above parameters is dynamic light scattering (DLS). DLS uses the deconvolution of a correlation function for the scattering intensity to deduce a size distribution spectrum based on signal intensity (or a calculated size distribution based on volume or number of particles). While SEM analysis can provide key insights into the “as manufactured” state, DLS provides insights into the in situ state of the particles.

Here, SPNP images (SEM) and property results (size and geometric factors) are presented in Figure 1b and Supporting Information File 1, Table S1. A dry-state size trend of TF > HSA > MUC ≈ HEM > INS can be observed when considering mean, median, and interquartile range (IQR) values. Direct inspection of the minimum diameter also reveals a similar trend of TF (65 nm) > HSA > HEM ≈ MUC ≈ INS (43 nm). Comparable PDI_SEM_ values can be observed for TF, MUC, HSA and INS (0.16–0.19); HEM is the most uniform (0.11) formulation. TF, HEM, and HSA have comparable anisotropy and roundness values, while MUC and INS possess increased anisotropy and lower roundness values. All single-protein SPNPs have high circularity (0.82–0.85). While variations in the properties of each single-protein SPNP system exist, SPNPs prepared from single proteins via EHD jetting are generally small (<100 nm), circular (>0.8), and uniform (SEM PDI_SEM_ < 0.2). To provide both quantitative and semi-quantitative comparisons of each blended particle with each of the monospecies particles, we undertook a comprehensive 2D multi-property analysis, which is presented in Tables S2–S5 and Figures S2–S5 of Supporting Information File 1.

### HEM/HSA-blended SPNPs

**Figure 2:**
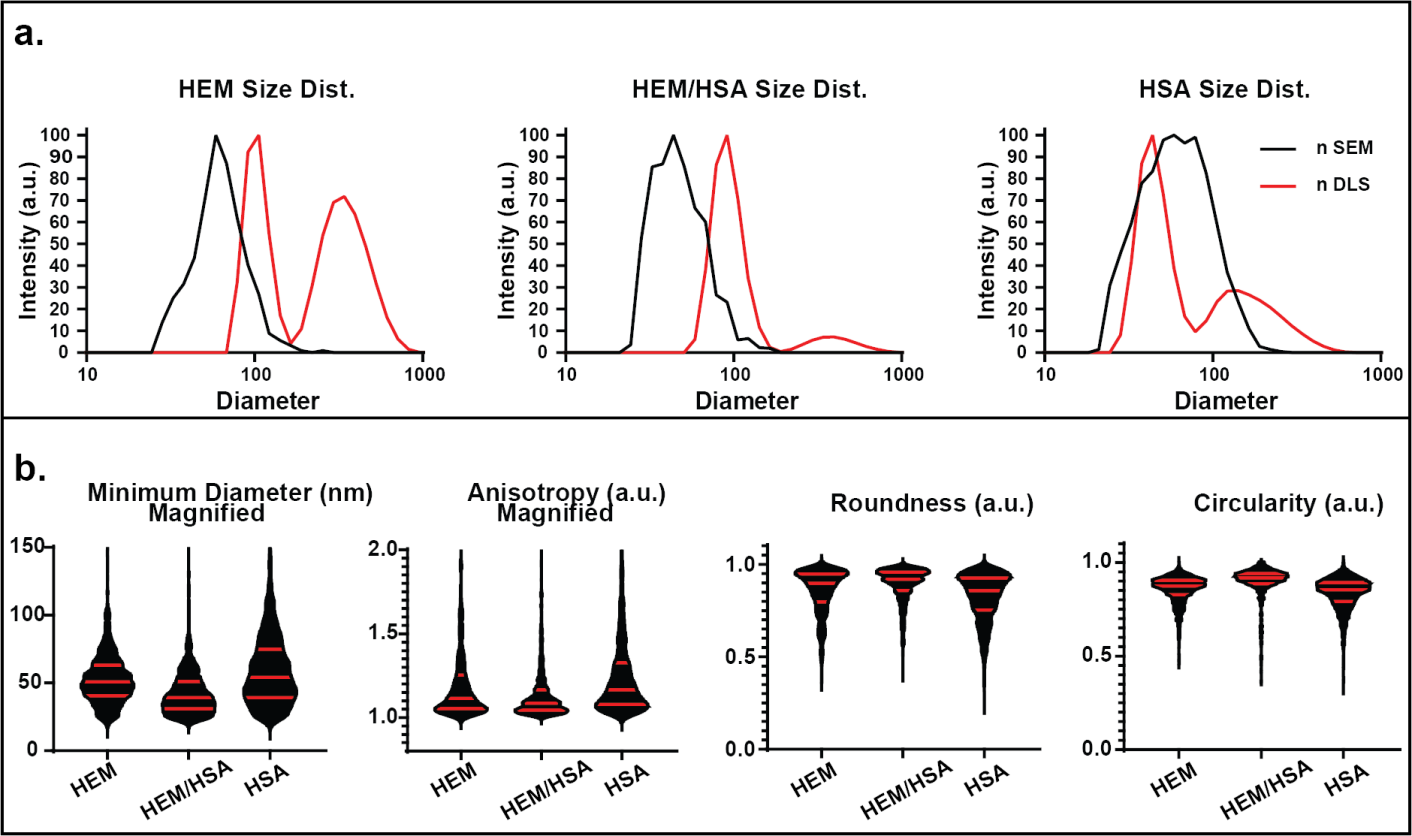
Size distribution and secondary geometric factors of HEM SPNPs based on SEM and DLS analysis. (a) Number distributions of SPNP sizes as obtained by SEM and DLS. (b) Violin graphs of minimum diameter, anisotropy, circularity and roundness (median and interquartile ranges are presented by red lines).

Both, the number average (nDLS) and nSEM distributions for the series of blended SPNPs are presented in Figure 2a. The HEM/HSA spectra indicate the presence of two populations, with a smaller diameter group as the dominant subpopulation (nDLS) and a broader/larger secondary subpopulation. When referring to DLS size results, a denotation of *d*_1_ was used to refer to the average of the smallest diameter distribution. Similarly, *d*_2_ refers to the average of any larger diameter distribution. When multiple subpopulations are assessed within a given nDLS distribution, deconvolution is utilized (Supporting Information File 1) to extract relative subpopulation fractions (α_1_, α_2_) (Table S6). The HEM/HSA population with *d*_1_ = 97 nm comprises swollen individual SPNPs, while the second population with *d*_2_ = 455 nm may be attributed to transient or semi-transient clusters. When compared to the HEM and HSA SPNPs, HEM/HSA SPNPs appear more similar to the sizes of HEM (*d*_1_ = 91 nm, *d*_2_ = 347 nm) than HSA nanoparticles (*d*_1_ = 46 nm, *d*_2_ = 222 nm). The results for these subpopulations are presented in Supporting Information File 1, Table S6. These findings are corroborated by the SEM results that found for HEM/HSA SPNPs a right-skewed population in the 30–100 nm diameter range (*d* = 51 nm), which is much more aligned with the monomodal HEM distribution (*d* = 65 nm) than the broader HSA distribution (*d* = 77 nm).

Geometric factors (minimum diameter, circularity, anisotropy and roundness) were assessed by SEM analysis (Figure 2b) in order to further elucidate how the shapes of the SPNPs are affected by the choice of protein. Statistical comparisons between the distributions are provided in Supporting Information File 1, Table S2. When considering these factors, the circularity can be thought of as an approximation of circle-like shape, the anisotropy is the ratio between major and minor diameter, and the roundness is an factor independent on the aspect ratio that describes edge smoothness [31–33].

In addition, the HEM/HSA minimum diameter distribution resembles more closely that of HEM SPNPs, while being smaller than both the individual HEM and HSA SPNPs. Similar to the minimum diameters, the anisotropy is both less variable and smaller (avg. = 1.14, Q1/Med./Q3 = 1.04/1.09/1.17) for blended SPNPs than for HEM (avg. = 1.20, Q1/Med./Q3 = 1.05/1.11/1.25) and HSA (avg. = 1.25, Q1/Med./Q3 = 1.00/1.17/1.33) particles (Figure 2b). This indicates that intentional formulation of blended SPNPs with appropriate protein ratios may generate features outside of the range obtained by single-protein SPNPs. Circularity follows the trends observed for other geometric factors: HEM/HSA SPNPs mimic HEM more than HSA particles, while possessing a higher circularity and lower variance than either of those. Inspection of roundness, similar to other parameters, appears to indicate that blending of proteins resulted in roundness values closer to 1 with smaller IQRs (avg. = 0.89, Q1/Med./Q3 = 0.86/0.92/0.96) when compared to HEM (avg. = 0.86, Q1/Med./Q3 = 0.80/0.90/0.95) and HSA (avg. = 0.82, Q1/Med./Q3 = 0.75/0.86/0.93). Given that all shape factors indicate that HEM/HSA SPNPs are more controlled, smaller, and more symmetrical, blending may be an effective strategy for modulating the diameter/minimum diameter of SPNPs.

To better understand how the SPNP populations might express secondary geometric factors, HEM/HSA SPNPs were also assessed via two-factor analysis. For each two-factor plot presented in Supporting Information File 1, Figure S2, the diameter of each particle is paired with a geometric factor for that particle. The similarities between these two-factor plots are given a score based on the degree to which HEM/HSA mimics either of the two individual SPNP distributions (Supporting Information File 1, Table S2). The HEM/HSA diameter–minimum diameter relationship is moderately (2.2) governed by hemoglobin. The diameter–anisotropy relationship has a minor (0.7) similarity to HSA SPNPs. These types of relationships are not assessable when only single parameters are considered. HEM/HSA diameter–circularity saw a minor (0.4) similarity to HEM, while the diameter–roundness relationship was moderately (1.6) similar to that of the HSA SPNPs. However, none of the two-factor comparisons were statistically predictive.

### TF/HSA blended SPNPs

**Figure 3:**
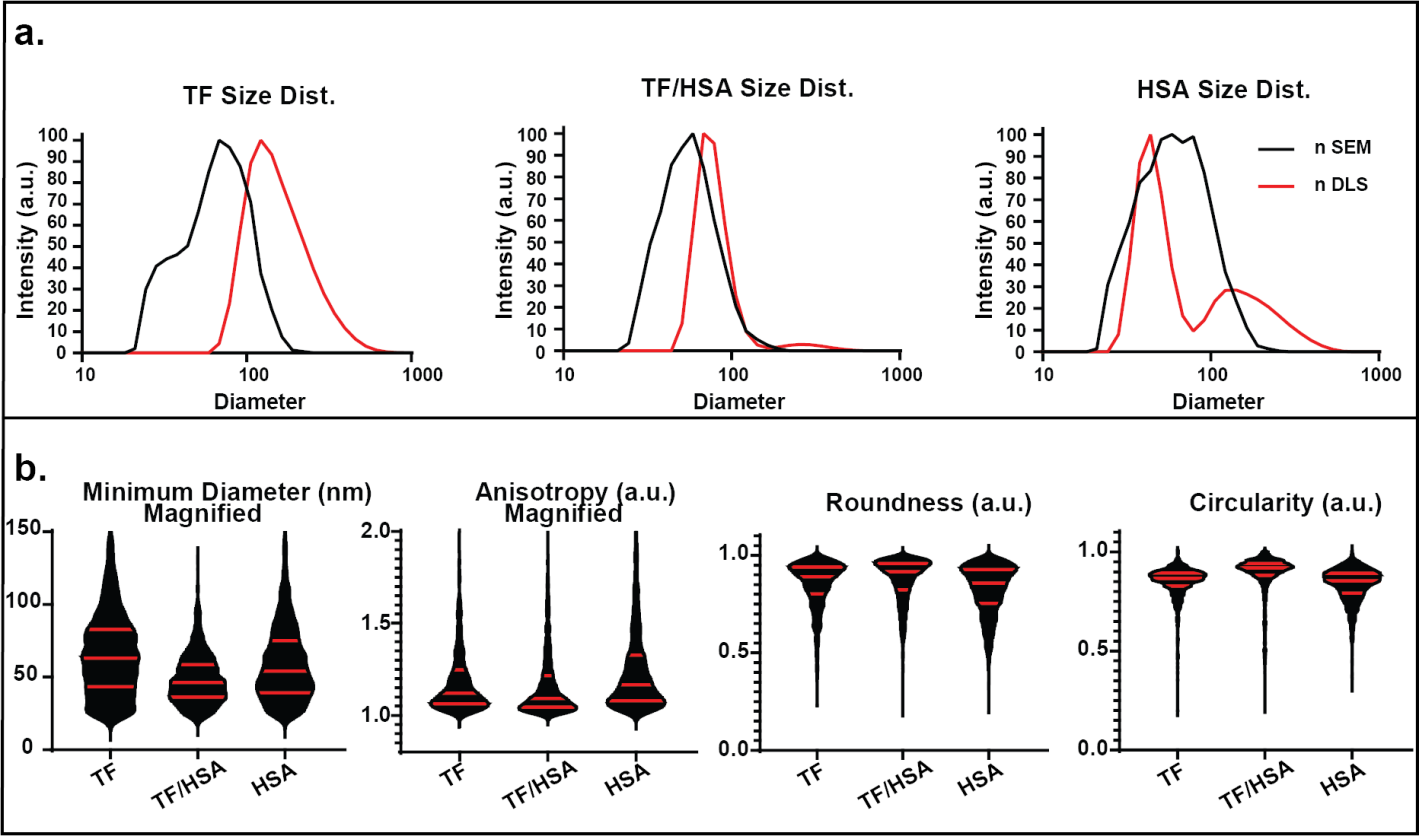
Size distribution and secondary geometric factors of TF SPNPs based on SEM and DLS analysis. (a) Number distributions of SPNP sizes as obtained by SEM and DLS. (b) Violin graphs of minimum diameter, anisotropy, circularity and roundness (median and interquartile ranges are presented by red lines).

The nDLS and nSEM data are presented in Figure 3a. The nDLS spectra for TF/HSA indicate the existence of two subpopulations, which were attributed to individual particles (*d*_1_ = 80 nm) and larger SPNP clusters (*d*_2_ = 326 nm). Compared to the single-component SPNPs, the value of *d*_1_ (80 nm) falls between HSA SPNPs (*d*_1_ = 46 nm) and TF SPNPs (*d*_1_ = 125 nm) (Supporting Information File 1, Table S6).

In the dry state, TF/HSA SPNPs display an average nSEM diameter (*d* = 59 nm) that is smaller than that of both HSA (*d* = 77 nm) and TF (*d* = 81 nm) with a narrower size distribution, as confirmed by a lower SEM PDI_SEM_ (0.16). The iSEM data indicates the same trend; TF/HSA is smaller (*d* = 92 nm) than TF (*d* = 109 nm) and HSA SPNPs (*d* = 116 nm) (Supporting Information File 1, Table S1). Taken together, these results indicate that dry TF/HSA SPNPs are smaller than TF and HSA in the dry state, but swell to an average diameter between those of the single-protein SPNPs.

For the TF/HSA series, the geometric factors are presented in Figure 3b, Figure S3, and Table S3 (Supporting Information File 1). Similar to the HEM/HSA SPNPs, the TF/HSA minimum diameter distribution was smaller and had lower variance than the distributions of TF and HSA SPNPs. For TF/HSA SPNPs, the sizes, anisotropy, and circularity of the system (as well as their variances) indicate a system that is more controlled, smaller, and more symmetrical than the single-protein SPNPs. These results further reinforce that protein blending can be a path to improved control of factors such as monodispersity and aspect ratio. The anisotropy of TF/HSA SPNPs (avg. = 1.17, Q1/Med./Q3 = 1.04/1.09/1.22) is more similar to TF (avg. = 1.21, Q1/Med./Q3 = 1.06/1.12/1.25) than HSA (avg. = 1.25, Q1/Med./Q3 = 1.08/1.17/1.33). TF/HSA SPNPs are more circular (avg. = 0.89, Q1/Med./Q3 = 0.88/0.92/0.94) than TF (avg. = 0.85, Q1/Med./Q3 = 0.83/0.87/0.89) and HSA (avg. = 0.83, Q1/Med./Q3 = 0.79/0.85/0.89) SPNPs (Figure 3b). TF/HSA roundness (less impacted by anisotropy than circularity) is similar to that of TF, though with less variance. Accordingly, the TF/HSA diameter–minimum diameter relationship indicates a strong bias towards HSA-like behavior (score = 8.2). The diameter–anisotropy relationship appears to be moderately governed by the more hydrophobic HSA (2.1). While the TF/HSA diameter–circularity similarity to HSA appears minor (0.8), there is a moderate similarity between the diameter–roundness relationships (2.7) (Supporting Information File 1, Figure S3, Table S3). This indicates that the transferrin contribution to the properties of blended SPNPs appears to be eclipsed by the more hydrophobic HSA component.

### MUC/HSA SPNPs

**Figure 4:**
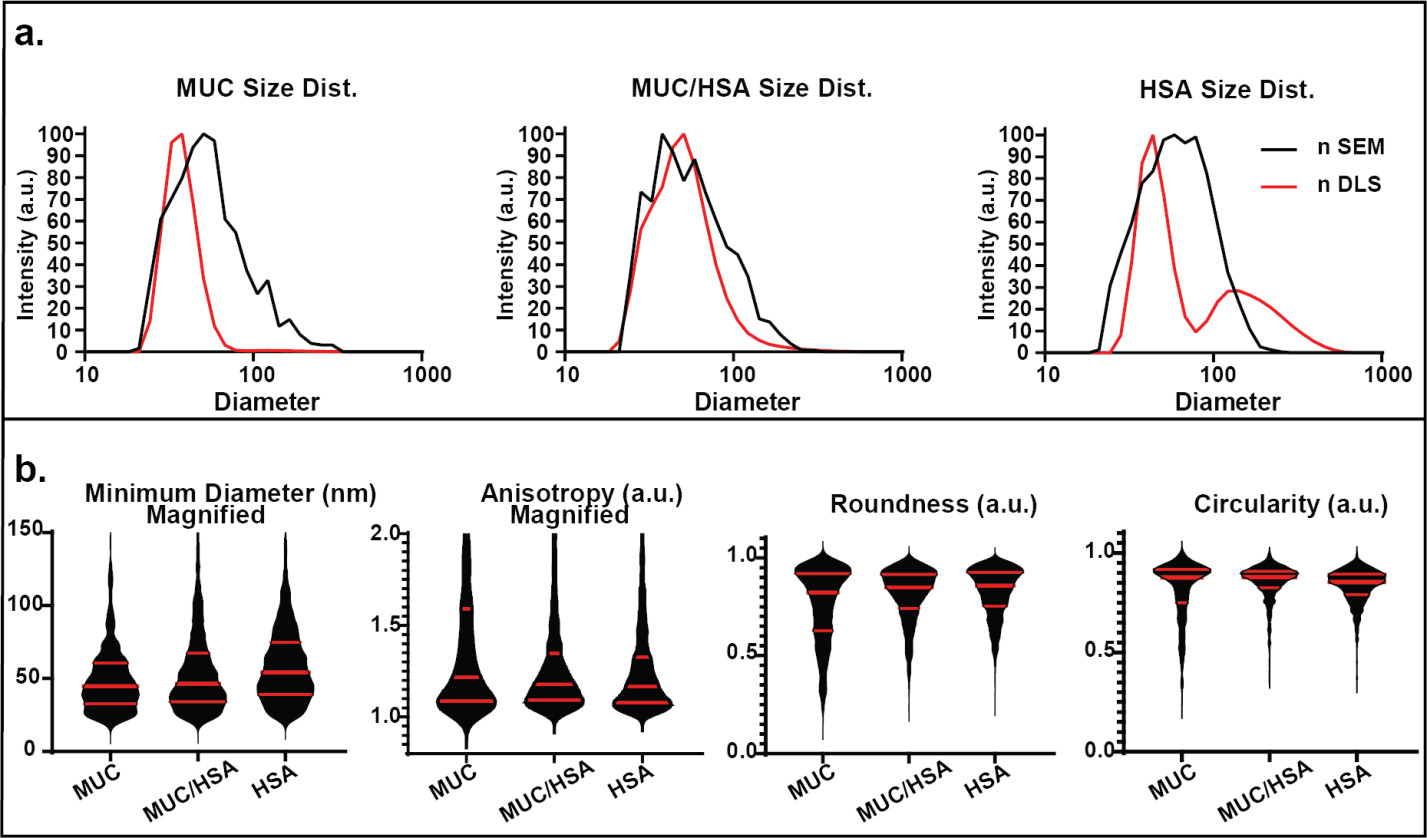
Size distribution and secondary geometric factors of MUC SPNPs based on SEM and DLS analysis. (a) Number distributions of SPNP sizes as obtained by SEM and DLS. (b) Violin graphs of minimum diameter, anisotropy, circularity and roundness (median and interquartile ranges are presented by red lines).

DLS and SEM results for MUC/HSA SPNPs are presented in Figure 4a. After deconvolution, the nDLS spectrum of MUC/HSA SPNPs displays a minor fraction (*d*_1_ = 30 nm, α_1_ = 0.07) and a dominant fraction at *d*_2_ = 68 nm. When compared to MUC and HSA SPNPs, MUC (*d*_1_ = 39 nm, *d*_2_ = 180 nm), not HSA (*d*_1_ = 46 nm, *d*_2_ = 222 nm), particles more closely resemble the blended MUC/HSA SPNPs (Supporting Information File 1, Table S6).

The nSEM analysis of MUC/HSA SPNPs reveals size distributions similar to both MUC (*d* = 73 nm) and HSA (*d* = 77 nm) SPNPs. The iSEM data suggest that MUC/HSA (*d* = 138 nm) SPNPs fall between MUC (*d* = 168 nm) and HSA (*d* = 116 nm) particles (Supporting Information File 1, Table S1). For both MUC and MUC/HSA, a significant overlap of the populations for the nSEM and nDLS distributions can be observed (Figure 4a). Taken together, these results indicate that the MUC/HSA particle sizes are governed by the hydrophilic mucin. We note that mucin is the only protein with a lower isoelectric point (IP = 2.75) than HSA (IP = 4.7). A detailed assessment of geometric features is provided in Figure 4b, Figure S4, and Table S4 (Supporting Information File 1). Assessment of the minimum diameter indicates that MUC/HSA appears to more closely resemble MUC than HSA. However, the anisotropy of MUC/HSA SPNPs appears to be equally influenced by both the HSA and the MUC components. Although the average circularity of all three SPNP formulations is similar (0.82–0.85), MUC/HSA SPNPs possess smaller IQRs and have lower variance. The roundness of MUC/HSA SPNPs (avg. = 0.81, Q1/Med./Q3 = 0.74/0.85/0.92) rests between those of MUC SPNPs (avg. = 0.76, Q1/Med./Q3 = 0.63/0.82/0.92) and HSA (avg. = 0.82, Q1/Med./Q3 = 0.75/0.85/0.93) (Figure 4b). The diameter–minimum diameter relationship of MUC/HSA SPNPs appears to strongly mimic the HSA SPNP relationship (8.9), as does the diameter–anisotropy relationship (7.9). The diameter–circularity (4.6) and diameter–roundness (7.2) relationships for MUC/HSA are also aligned with the HSA response (Supporting Information File 1, Figure S4, Table S4). These relationships between secondary geometric attributes and particle diameter indicate that the two-factor response of MUC/HSA is dominated by the more hydrophobic HSA constituent, a fact that was not evident from assessment of individual SPNPs alone.

### INS/HSA SPNPs

**Figure 5:**
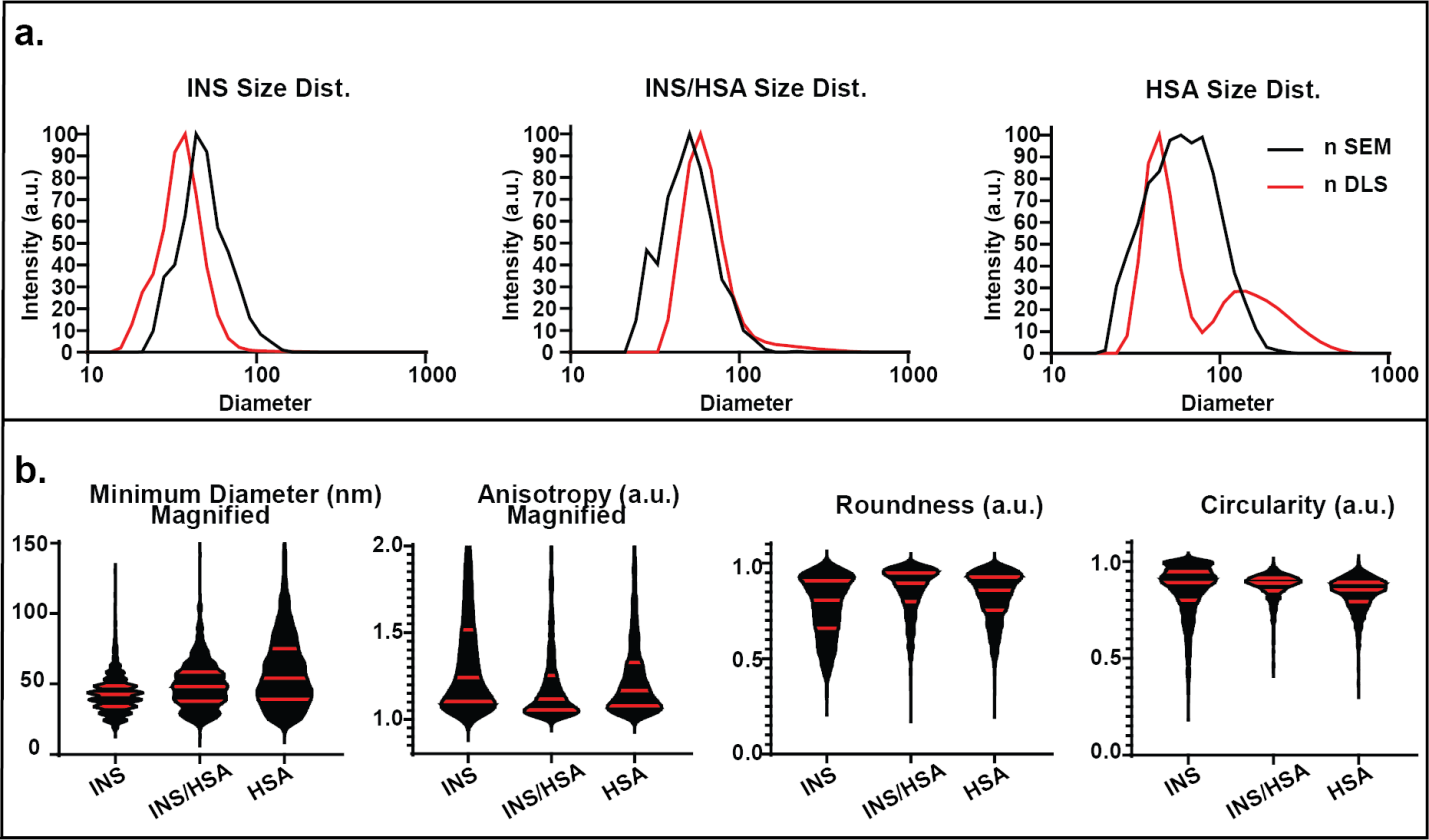
Size distribution and secondary geometric factors of INS SPNPs based on SEM and DLS analysis. (a) Number distributions of SPNP sizes as obtained by SEM and DLS. (b) Violin graphs of minimum diameter, anisotropy, circularity and roundness (median and interquartile ranges are presented by red lines).

The DLS and SEM analysis of INS/HSA SPNPs are presented in Figure 5a. INS/HSA SPNPs display two populations with nDLS diameter of *d*_1_ = 64 nm and *d*_2_ = 138 nm.

When compared to INS (*d*_1_ = 35 nm, *d*_2_ = 144 nm) and HSA (*d*_1_ = 46 nm, *d*_2_ = 222 nm) the average size of INS/HSA SPNPs is significantly larger, an effect not observed for the other blended SPNPs (Supporting Information File 1, Table S6). The nSEM distribution of INS/HSA SPNPs (*d* = 61 nm) more closely aligns with those of INS SPNPs (*d* = 60 nm) and is clearly different from the larger and broader distribution of HSA SPNPs (*d* = 77 nm). Similar to the other blended SPNPs, the SEM diameter distribution is less influenced by HSA. The iSEM data of INS/HSA SPNPs (*d* = 83 nm) displays a deviation from the INS population (*d* = 49 nm), with a broader distribution more similar to that of HSA SPNPs (*d* = 116 nm) (Supporting Information File 1, Table S1). The results from SEM and DLS indicate that while dry INS/HSA SPNPs are more similar in size to INS they increase in size and variance in the swollen state, which approaches a HSA SPNP-like distribution.

The analysis of geometric factors is presented in Figure 5b and Figure S5; the results are presented in Supporting Information File 1, Table S5. The minimum diameter results of INS/HSA SPNPs lie between the distributions observed for the individual SPNPs, whereas the anisotropy of INS/HSA SPNPs is biased towards a HSA-like distribution. However, both the average and IQRs for the anisotropy of the blended SPNPs is lower than those of either INS or HSA (an effect also observed in other blended SPNPs). The average circularity of INS/HSA SPNPs resembles that of INS SPNPs. Lastly, the INS/HSA system presents as rounder than either of the other systems according to all key metrics (average, IQRs, and variance) (Figure 5b). The properties of INS/HSA SPNPs appear to be a mix of responses guided by both proteins. For comparison, the two-factor results for INS/HSA SPNPs can be found in Figure S5 and Table S5 (Supporting Information File 1). The diameter–minimum diameter relationship appears to mirror both HSA (score = 7.0) and INS (score = 1.8) SPNPs. The diameter–anisotropy relationship of INS/HSA SPNPs has a moderate agreement with both HSA (4.5) and INS (2.5) particles. HSA SPNPs strongly align with the INS/HSA response for diameter–circularity (7.9), but appear to have very little in common with INS (0.1). The diameter–roundness relationship of INS/HSA SPNPs is a combination of the factors observed in HSA (3.6) and INS (3.5).

## Conclusion

In this work, we expand the conceptual framework of SPNP nanocarriers in a systematic way, while exploring the relationships between blended-protein and single-protein systems in both dry and hydrated states. We have shown that tailoring formulations of SPNPs through blending and/or protein selection (Supporting Information File 1, Table S7) can result in a robust platform for exploring a variety of sizes, uniform size distributions, and shape parameters. One-factor and two-factor SEM analyses, combined with DLS, revealed that most of the blended SPNPs have geometric parameters that align with one of the constituent proteins. Those blended SPNPs may have predictable physical properties that can be tuned by changing their respective protein-to-protein ratios. Future efforts will include the application of these methods to a range of nanomedicine applications and further studies aimed at correlating these properties with SPNP performance in vitro and in vivo. The direct scalability of the electrohydrodynamic co-jetting method will likely involve setups with multiple parallel jets [34].

## Experimental

### Materials

Recombinant human serum albumin was purchased from InVitria. Mucin from porcine stomach, human transferrin, human hemoglobin, recombinant human insulin, and *O*,*O*′-bis[2-(*N*-succinimidylsuccinylamino)ethyl]polyethylene glycol (NHS-PEG-NHS) with a molecular weight of 2000 Da were purchased from Sigma-Aldrich, USA. *O*,*O*′-bis[2-(*N*-succinimidyl-succinylamino)ethyl]polyethylene glycol (NHS-PEG-NHS) with a molecular weight of 400 Da was purchased from Nanocs Inc., USA.

### EHD jetting

EHD jetting followed the protocol published by Rahmani and co-workers [35]. Briefly, a protein solution was pumped at 0.1 mL/h through a 25 Ga needle. Once an electric voltage was applied, the meniscus was distorted and formed a Taylor cone [25]. An electrically charged protein solution then ejected from the apex of the Taylor cone, directed to a grounded substrate positioned below the cone. The ejected material dissociated into nanodroplets. After rapid solvent evaporation and solidification of non-volatile components, solid nanoparticles were deposited on the substrate. The reaction between lysine groups and NHS ester groups of the macromer resulted in chemical gelation, which was initiated during the jetting and allowed to continue for another 7 days at 37 °C in order to complete the reaction and ensure that there were no remaining NHS ester groups. The voltage biases ranged between 8 and 10 kV in all instances and were only changed between runs based on Taylor cone stabilization. The biases were generated from positive lead attachment to the jetting needle and ground lead attachment to the collection plate.

### SPNP formulations

For SPNPs, the protein of interest was dissolved at 10% (w/v) in a solvent mixture of water and ethanol with a 9:1 (v/v) ratio. The exception was insulin and mucin-based SPNPs. Because insulin has poor solubility at neutral pH, acetic acid at 10% (v/v) was added to the solvent mixture to ensure miscibility. Due to its high molecular weight, mucin was used at 2% (w/v). NHS-PEG-NHS macromer with a molecular weight of 400 Da was added to the solution at 10% (w/w_protein_) relative to the protein solution. For all blended SPNPs, a 1:1 (w/w) protein mixture was maintained, where the second protein was always HSA, except in the mucin/HSA system, where 4% (w/v) mucin was used. A table detailing the exact masses and volumes for these formulations is provided in Supporting Information File 1 (Table S8).

### Scanning electron microscopy

SEM images were obtained using a FEI Nova 200 Nanolab SEM/FIB at the Michigan Center for Materials Engineering using an acceleration voltage of 5 kV. Particles were sputter coated with gold for 40 s using an SPI-Module Carbon/Sputter Coater, which is optimized for monolayer deposition. Typical fields of view (FOV) were 5 μm and pixel sizes were in the range of 2 nm. Collected images were semi-randomized; a random FOV was selected approximately near the center of mass for the substrate, with subsequent images taken at a set distance in each cardinal direction (+*X*, −*X*, +*Y*, −*Y*) in order to avoid bias. The SEM images were taken from a silicon wafer that was obtained from direct placement onto the substrate during jetting. This wafer was then sputter coated with gold prior to evaluation in the SEM system. Care was taken to not overexpose the sample during gold preparation, as evidenced by a lack of melted particles or crevice filling in the final micrographs.

The SEM PDI is a polydispersity index derived from image generated data. The method by which it is calculated is comparable to PDI values generate from DLS insomuch as it is the standard deviation of a calculated volume distribution divided by the average sizes produced by the volume calculated distribution. This emulates the SD/z-avg methodology utilized in DLS, and also provide a reasonable metric for the comparison of various SEM distributions.

### Collection and processing of SPNPs

The SPNP collection process followed standardized protocols previously described [25]. Briefly, a 2 mL solution of 0.01% of Tween20 in Dulbecco's phosphate-buffered saline (DPBS) was added to the crosslinked SPNPs collection plates and physically agitated to release the SPNPs from the surface of the collection plates. This suspension was sonicated to disrupt SPNPs aggregates then filtered through a 40 µm cell filter to remove any large debris. To further remove larger SPNPs, undisrupted aggregates and other debris, the following centrifugation steps were followed. First, the SPNPs were centrifuged at 3220 rcf for 5 min whereby the pellet was discarded and the supernatant was further centrifuged at 21500 rcf for 1 h at 10 °C. The final SPNPs were washed with DPBS to remove remaining Tween20 used in the collection process.

### Dynamic light scattering

DLS measurements were performed on particles in their hydrated state using a Zetasizer Nano ZS (Malvern Panalytical). The solution in which the particles were suspended was DPBS. DLS was employed to measure the particle size distribution after particle collection and serial centrifugation (performed to eliminate any large or anomalous structures that are known to compromise DLS results). The average of at least three measurements was reported (*n* > 3). The duration of each scan was between 60 and 80 s. The approximate concentration of particles, as measured during DLS, is 10^10^ to 10^11^ particles per milliliter for particles with an average diameter of 200 nm. Attenuation was kept to a minimum (50% at most) in order to keep CPS within the operation range of the instrument. Specifically, the range of the count rates compared was from 120–473 kcps (avg. 231 kcps, sd 90 kcps). DLS results are the application of the Mark–Houwink equations within the software (Malvern Zetasizer Software 8.01.4906). The results generated from this makes assumptions regarding the intrinsic viscosity, which can only be an estimate since the local viscosity in polymer and/or protein nanoparticles will vary on a per particle basis. Additionally, the presumption of a molecular weight/diameter equivalency in these models may not be the most appropriate for assessing nanogel systems.

### Statistical analysis

Statistical analyses were performed using Graphpad, Prism 9.0.0, (GraphPad Software, LaJolla, CA). Analysis of variance, followed by Tukey’s post-test was used. Non-paired, two-tailed *t*-tests were used. A *P*-value below 0.05 was considered statistically significant (*P < 0.05, **P < 0.01, ***P < 0.001; ****P < 0.0001); *P*-values above 0.05 were considered not significant (ns). Additional details are provided in Supporting Information File 1.

### Analysis and nomenclature

The analysis of SEM is presented as nSEM distributions (individuals data from *n* > 1000 particles). In parallel to SEM size analysis, key geometric factors were also extracted. These included minimum diameter, anisotropy, circularity, and roundness. For two-factor analysis, a similarity score for comparing the blended SPNPs to single-protein SPNPs are reported. Score values are assessed as follows: A score of 0 indicates the system is not discernably impacted by a constituent relative to the other constituent; a score of >0 and ≤1 indicates that a minor impact is observed; a score of >1 and ≤5 indicates a moderate impact is observed; and a score of >5 indicates a major impact. The calculation of this score is derived from comparison of linear regression fits as outlined in Supporting Information File 1. While the majority of the discussion is framed in terms of number average results, all (nSEM, nDLS, iSEM, iDLS) distribution data can be found as summary results (Supporting Information File 1, Table S1). Here, nSEM data are the histograms of raw SEM micrograph analysis for individual particles binned for comparison to nDLS; nDLS is the number DLS spectra obtained directly from the instrumentation and should be compared directly to nSEM data; iSEM represents a volumetric transform of the SEM data into histograms that are appropriate for comparison to the iDLS spectra; iDLS is the intensity DLS spectra obtained directly from the instrumentation and should be compared directly to iSEM data.

## Supporting Information

File 1 Additional details.
