# Supplementary material for: Systematic studies into uniform synthetic protein nanoparticles
Source: Beilstein J Nanotechnol. 2022 Feb 28;13:274–83. doi: 10.3762/bjnano.13.22 (PMC8919420; doi:10.3762/bjnano.13.22)
Supplement: File 1 — Additional details. [file Beilstein_J_Nanotechnol-13-274-s001.pdf]

BEILSTEIN JOURNAL OF NANOTECHNOLOGY

Supporting Information

for

Systematic studies into uniform synthetic protein nanoparticles

Nahal Habibi, Ava Mauser, Jeffery E. Raymond and Joerg Lahann

Beilstein J. Nanotechnol. **2022,** *13,* 274–283. doi:10.3762/bjnano.13.22

Additional details

License and Terms: This is a supporting information file under the terms of the Creative Commons Attribution License (https://creativecommons.org/licenses/by/4.0). Please note that the reuse, redistribution and reproduction in particular requires that the author(s) and source are credited and that individual graphics may be subject to special legal provisions.
The license is subject to the *Beilstein Journal of Nanotechnology* terms and conditions: (https://www.beilstein-journals.org/bjnano/terms)

1 Instrumentation

1.1 Scanning electron microscopy

The SEM micrographs presented in this article are cropped and magnified from larger fields of view for clarity and ease of particle observation. Figure S1 provides full field of view of the typical images utilized to build population statistics throughout the study.

1.2 Dynamic light scattering

The particle size distributions were measured with dynamic light scattering (Malvern ZSP ZEN-5600). The protein material was chosen with refractive index of 1.45 and absorbance of 0.001. Phosphate-buffered saline was used as the dispersant with refractive index of 1.332 and viscosity of 0.9074. Measurements were done at 25 °C, at 3.00 mm position in a disposable microcuvette. (ZEN0040).

1.3 Analysis software

FIJI (a distribution of ImageJ v1.53c) was used for all image analysis. OfficeLibre Calc was used for all data manipulation and for the generation of summary statistics. Graphpad Prism 9.0 was used for presentation of distributions, scatter plots, and violin plots. Origin 9 was used for peak extraction.

2 SEM Analysis via ImageJ/FIJI

SEM images are collected as lossless TIFF files and were processed via ImageJ.

3 Data processing via open source spreadsheet software

In order to obtain results that will allow for comparison of the data from imaging with DLS-based PDI results for the systems, histograms and individual data were calculated as volume-based values in order to generate a calculated SEM-based PDI value (denoted here as PDI_{SEM}) and to present an intensity-based analog (iSEM) for comparison to intensity-based DLS. iSEM distributions, which were calculated from nSEM × $(d/2)^3$ and then normalized. This was done to provide dry particle (as manufactured) analysis that is comparable to the nDLS and iDLS results (as used in solution after post-processing). The entirety of the analysis for this step was done utilizing OfficeLibre Calc. The various expressions of diameter populations were evaluated using statistical analysis including t-test, IQR, and ANOVA.

4 Two-dimensional analysis

To understand how the geometric characteristics for blended SPNPs are influenced by their constituents, we quantitatively and qualitatively described the SPNPs. The diameter was compared to other geometric attributes (min. diameter, anisotropy, circularity, and roundness). The linear regressions of the paired *x*–*y* data sets were created and a scoring factor was used (from 0 to 10) to describe the extent of similarity to the monospecies SPNPs. For example, to understand how a blend of HSA and transferrin SPNP resemble their constituents, this scoring system can be applied. The scoring factor represents a convolution of the relative agreement of the blended regression slope and the agreement of the regression strength ($\langle r^2 \rangle$) when compared to the monospecies SPNPs. This is done by treating the slope of the blend regression as a linear combination of the slopes of the constituent regressions, scaling based on the extent of agreement between the strengths of the regressions.

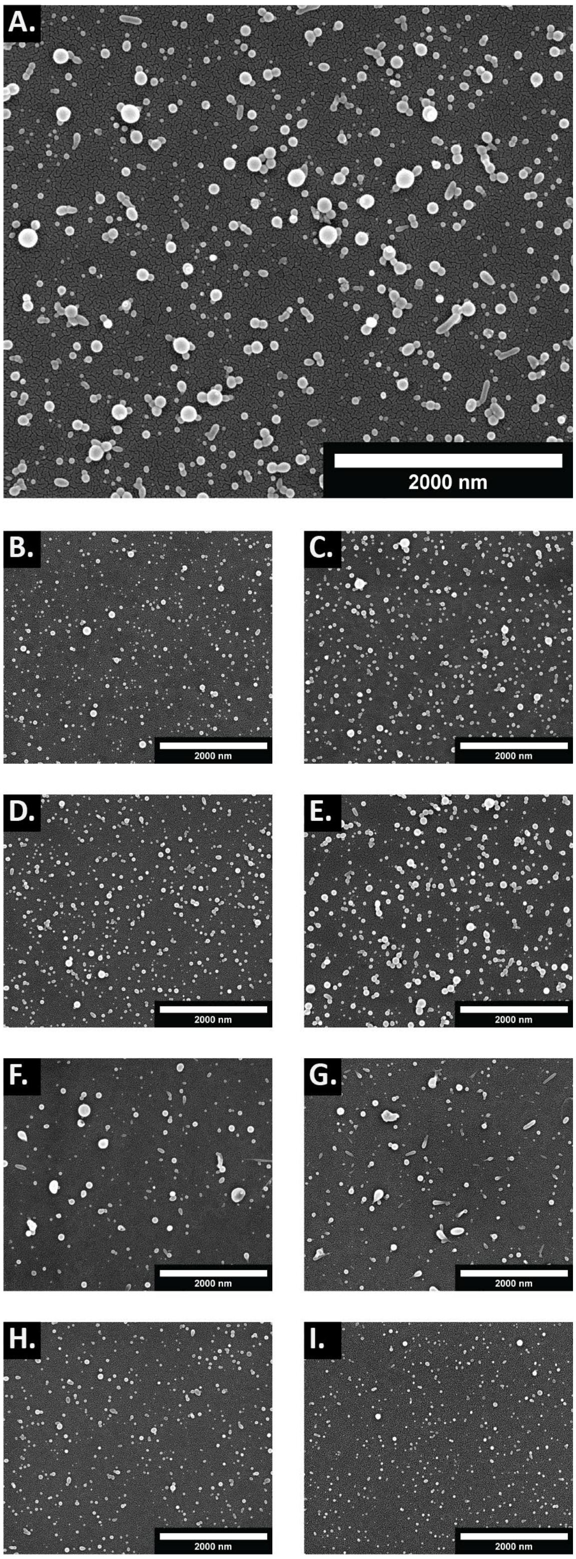

Figure S1: Large-area FOV SEM images of single-protein and blended SPNPs. (A) HSA, (B) HEM/HSA, (C) HEM, (D) TF/HSA, (E) TF, (F) MUC/HSA, (G) MUC, (H) INS/HSA, and (I) INS. Scale bars: 2000 nm

Table S1: SPNPs size and secondary geometric factors data. Average diameters are presented based on nSEM, nDLS, iSEM, and iDLS results. Minimum diameter, anisotropy, circularity, and roundness results are provided as secondary geometric factors.

**Formulation Name**	**Diameter, nSEM**	**Diameter, nDLS**	**Diameter, iSEM**	**Diameter, iDLS**	**PDI_{SEM} SEM**	**PDI iDLS**	**Min. Dia. SEM**	**Anisotropy SEM**	**Circularity SEM**	**Roundness SEM**
(units)	(nm)	(nm)	(nm)	(nm)	(a.u.)	(a.u.)	(nm)	(a.u.)	(a.u.)	(a.u.)
HEM	65 ± 25	254 ± 157	64 ± 12	382 ± 106	0.11	0.38	53 ± 19	1.20 ± 0.23	0.85 ± 0.08	0.86 ± 0.12
HEM/HSA	51 ± 20	127 ± 108	50 ± 15	475 ± 138	0.15	0.47	43 ± 17	1.14 ± 0.15	0.90 ± 0.09	0.89 ± 0.09
TF	81 ± 36	170 ± 84	109 ± 36	284 ± 124	0.19	0.22	65 ± 29	1.21 ± 0.24	0.85 ± 0.08	0.85 ± 0.12
TF/HSA	59 ± 23	86 ± 48	92 ± 36	328 ± 87	0.16	0.41	49 ± 17	1.17 ± 0.23	0.89 ± 0.11	0.87 ± 0.11
MUC	73 ± 45	39 ± 17	168 ± 79	262 ± 108	0.16	0.349	50 ± 25	1.45 ± 0.59	0.82 ± 0.15	0.76 ± 0.19
MUC/HSA	72 ± 42	55 ± 34	138 ± 61	270 ± 169	0.16	0.38	55 ± 30	1.28 ± 0.33	0.85 ± 0.09	0.81 ± 0.14
INS	60 ± 22	37 ± 15	49 ± 9	220 ± 82	0.17	0.468	43 ± 14	1.36 ± 0.35	0.85 ± 0.13	0.77 ± 0.16
INS/HSA	61 ± 23	70 ± 41	83 ± 38	269 ± 130	0.13	0.5	49 ± 17	1.20 ± 0.25	0.87 ± 0.07	0.86 ± 0.12
HSA	77 ± 37	97 ± 86	116 ± 44	283 ± 115	0.18	0.44	60 ± 27	1.25 ± 0.27	0.83 ± 0.09	0.82 ± 0.13

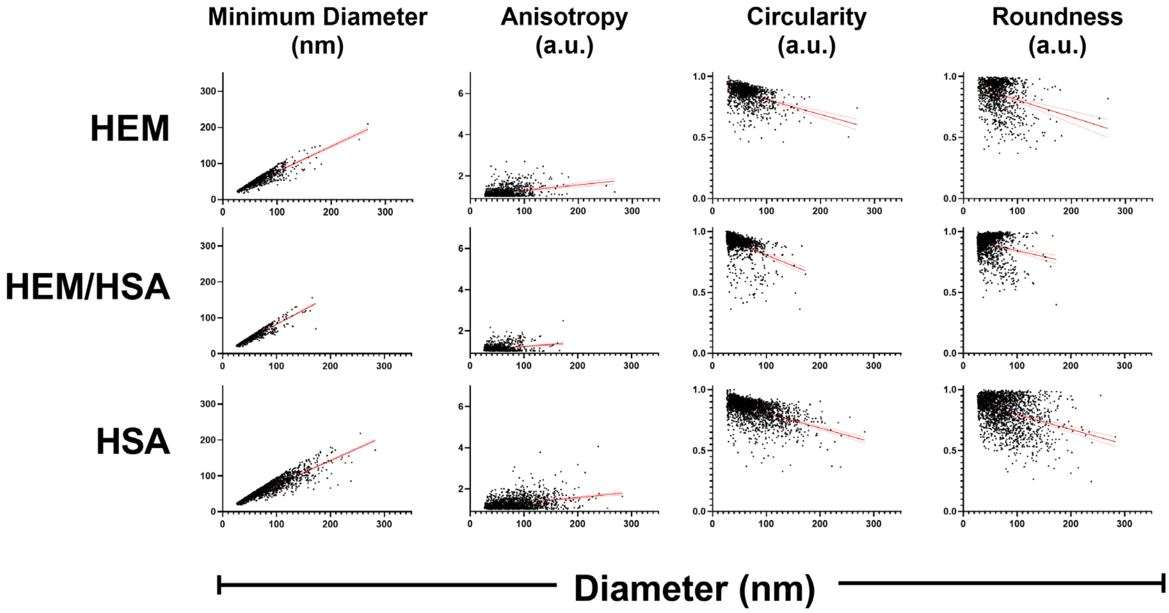

Figure S2: Two-factor individual analysis for the HEM series. Scatter plots of minimum diameter, anisotropy, circularity, and roundness vs diameter.

Table S2: HEM series statistical analysis results of secondary geometric factors and scoring data for two-factor analysis. Minimum diameter, anisotropy, circularity and roundness results are provided as secondary geometric factors.

SPNPs series	T-Test					Min. Dia			Anisotropy			Circularity			Roundness		
	Diameter	Min. Dia	Anisotropy	Circularity	Roundness	Slope	R^2	Score	Slope	R^2	Score	Slope	R^2	Score	Slope	R^2	Score
HEM	-	-	-	-	-	0.7008	0.8265	-	0.002715	0.08659	-	-0.001226	0.162	-	-0.001393	0.08027	-
vs.	$p < 0.0001$ ****	$p < 0.0001$ ****	$p < 0.0001$ ****	$p < 0.0001$ ****	$p < 0.0001$ ****	-	-	2.24077033	-	-	0	-	-	0.38718728	-	-	0
HEM/HSA	-	-	-	-	-	0.7939	0.8191	-	0.001965	0.06754	-	-0.001788	0.1645	-	-0.0009889	0.04516	-
vs.	$p < 0.0001$ ****	$p < 0.0001$ ****	$p < 0.0001$ ****	$p < 0.0001$ ****	$p < 0.0001$ ****	-	-	0	-	-	0.71602039	-	-	0	-	-	1.62334542
HSA	-	-	-	-	-	0.6736	0.8973	-	0.002611	0.1308	-	-0.001203	0.1308	-	-0.001203	0.1308	-
Three-Way	$p < 0.0001$ ****	$p < 0.0001$ ****	$p < 0.0001$ ****	$p < 0.0001$ ****	$p < 0.0001$ ****	-	-	-	-	-	-	-	-	-	-	-	-

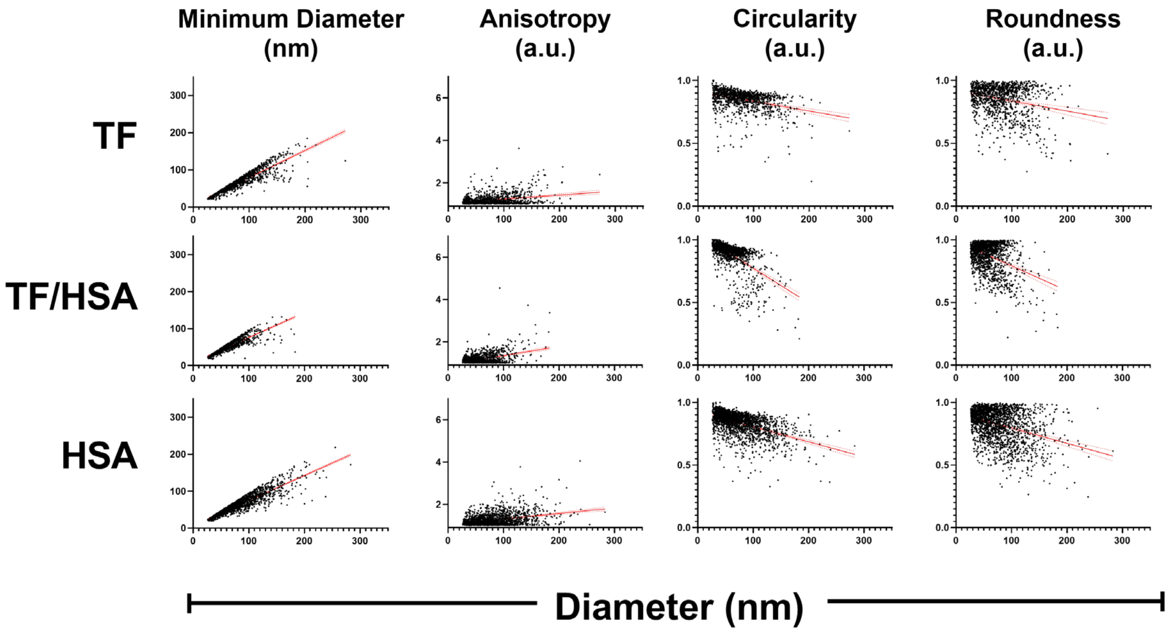

Figure S3: Two-factor individual analysis for the TF series. Scatter plots of minimum diameter, anisotropy, circularity, and roundness vs diameter.

Table S3: TF series statistical analysis results of secondary geometric factors and scoring data for two-factor analysis. Minimum diameter, anisotropy, circularity and roundness results are provided as secondary geometric factors.

SPNPs series	T-Test					Min. Dia			Anisotropy			Circularity			Roundness		
	Diameter	Min. Dia	Anisotropy	Circularity	Roundness	Slope	R^2	Score	Slope	R^2	Score	Slope	R^2	Score	Slope	R^2	Score
TF	-	-	-	-	-	0.7296	0.8393	-	0.001863	0.07776	-	-0.0007748	0.1298	-	-0.0008078	0.05926	-
vs.	p < 0.0001 ****	p < 0.0001 ****	p < 0.001 ***	p < 0.0001 ****	p < 0.0001 ****	-	-	0	-	-	0	-	-	0	-	-	0
TF/HSA	-	-	-	-	-	0.6694	0.795	-	0.004341	0.1888	-	-0.002779	0.361	-	-0.001994	0.1613	-
vs.	p < 0.0001 ****	p < 0.0001 ****	p < 0.0001 ****	p < 0.0001 ****	p < 0.0001 ****	-	-	8.2417796	-	-	2.09125046	-	-	0.77411618	-	-	2.70167058
HSA	-	-	-	-	-	0.6736	0.8973	-	0.002611	0.1308	-	-0.001203	0.1308	-	-0.001203	0.1308	-
Three-Way	p < 0.0001 ****	p < 0.0001 ****	p < 0.0001 ****	p < 0.0001 ****	p < 0.0001 ****	-	-	-	-	-	-	-	-	-	-	-	-

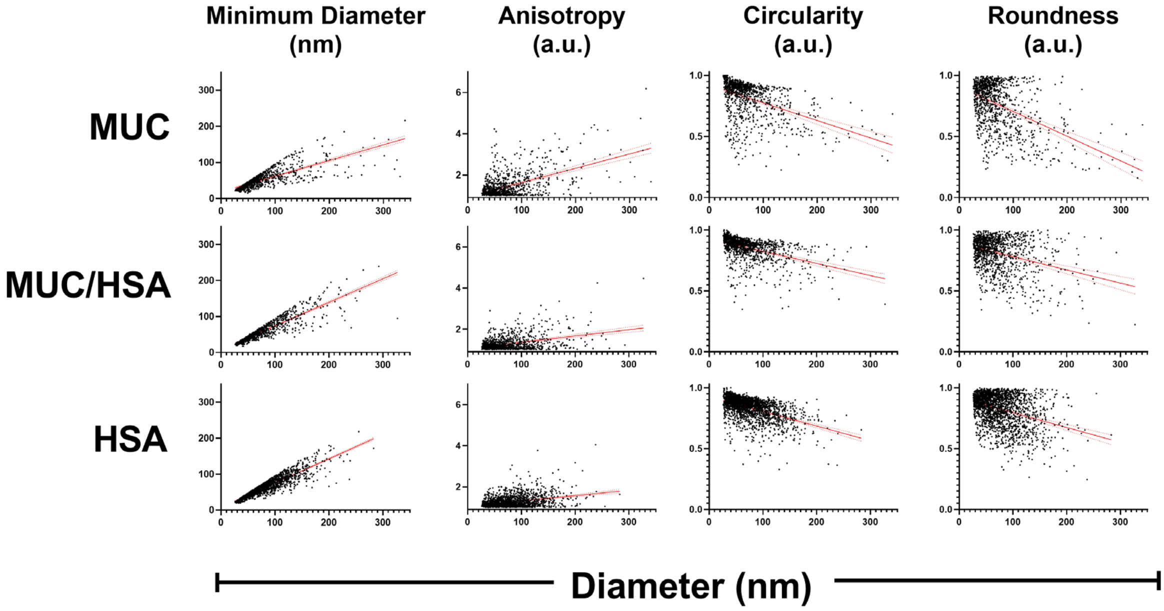

Figure S4: Two-factor individual analysis for the MUC series. Scatter plots of minimum diameter, anisotropy, circularity, and roundness vs diameter.

Table S4: MUC series statistical analysis results of secondary geometric factors and scoring data for two-factor analysis. Minimum diameter, anisotropy, circularity and roundness results are provided as secondary geometric factors.

**SPNPs series**	**T-Test**					**Min. Dia**			**Anisotropy**			**Circularity**			**Roundness**		
	**Diameter**	**Min. Dia**	**Anisotropy**	**Circularity**	**Roundness**	**Slope**	**R^2**	**Score**	**Slope**	**R^2**	**Score**	**Slope**	**R^2**	**Score**	**Slope**	**R^2**	**Score**
**MUC**	-	-	-	-	-	0.434	0.6186	-	0.006931	0.2886	-	-0.001442	0.2886	-	-0.002028	0.2308	-
**vs.**	ns	$p < 0.0001$ ****	$p < 0.0001$ ****	$p < 0.0001$ ****	$p < 0.0001$ ****	-	-	0.55790396	-	-	0.51046559	-	-	0	-	-	0
**MUC/HSA**	-	-	-	-	-	0.6559	0.8191	-	0.003035	0.1501	-	-0.0009936	0.1501	-	-0.001086	0.1073	-
**vs.**	$p < 0.01$ **	$p < 0.0001$ ****	$p < 0.01$ **	$p < 0.0001$ ****	$p < 0.05$ *	-	-	8.91934775	-	-	7.85890888	-	-	4.64471797	-	-	7.18447477
**HSA**	-	-	-	-	-	0.6736	0.8505	-	0.002611	0.1308	-	-0.001203	0.1308	-	-0.001203	0.1308	-
**Three-Way**	$p < 0.01$ **	$p < 0.0001$ ****	$p < 0.0001$ ****	$p < 0.0001$ ****	$p < 0.0001$ ****	-	-	-	-	-	-	-	-	-	-	-	-

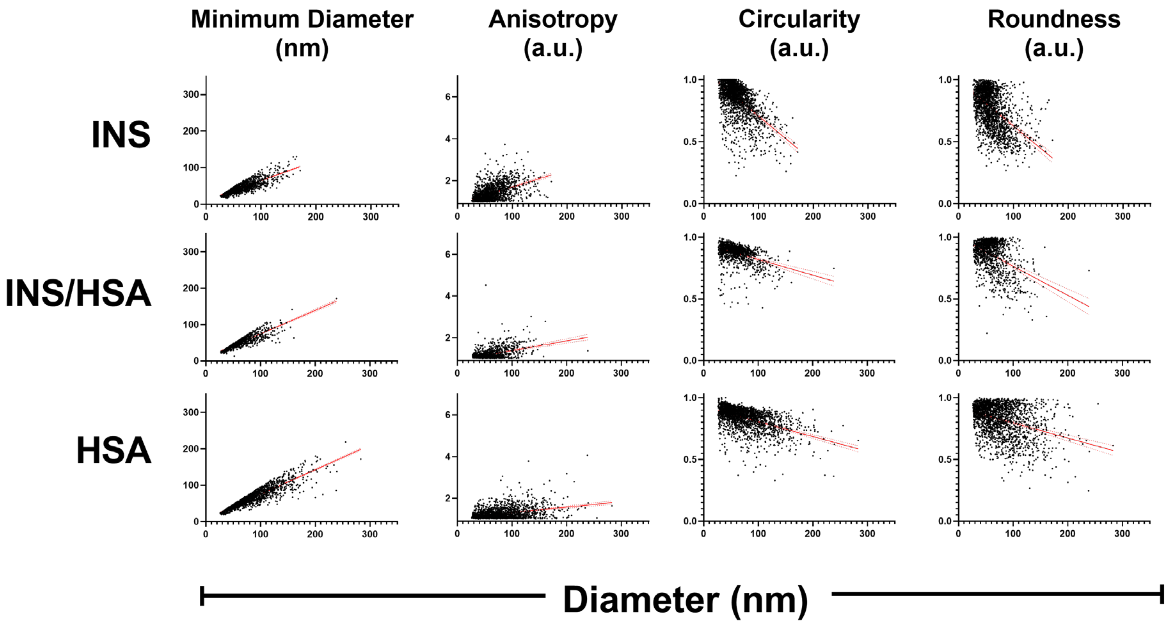

Figure S5: Two-factor individual analysis for the INS series. Scatter plots of minimum diameter, anisotropy, circularity, and roundness vs diameter.

Table S5: INS series statistical analysis results of secondary geometric factors and scoring data for two-factor analysis. Minimum diameter, anisotropy, circularity and roundness results are provided as secondary geometric factors.

SPNPs	T-Test					Min. Dia			Anisotropy			Circularity			Roundness		
series	Diameter	Min. Dia	Anisotropy	Circularity	Roundness	Slope	R^2	Score	Slope	R^2	Score	Slope	R^2	Score	Slope	R^2	Score
INS	-	-	-	-	-	0.5347	0.7088	-	0.008143	0.2598	-	-0.003668	0.3622	-	-0.003629	0.2598	-
vs.	ns	$p < 0.0001$ ****	$p < 0.0001$ ****	$p < 0.0001$ ****	$p < 0.0001$ ****	-	-	1.84428555	-	-	2.54632088	-	-	0.13286172	-	-	3.52396288
INS/HSA	-	-	-	-	-	0.6449	0.7941	-	0.004634	0.1809	-	-0.001277	0.1603	-	-0.002358	0.1923	-
vs.	$p < 0.0001$ ****	$p < 0.0001$ ****	$p < 0.0001$ ****	$p < 0.0001$ ****	$p < 0.0001$ ****	-	-	7.02128945	-	-	4.58638358	-	-	7.91474403	-	-	3.56355293
HSA	-	-	-	-	-	0.6736	0.8973	-	0.002611	0.1308	-	-0.001203	0.1308	-	-0.001203	0.1308	-
Three-Way	$p < 0.0001$ ****	$p < 0.0001$ ****	$p < 0.0001$ ****	$p < 0.0001$ ****	$p < 0.0001$ ****	-	-	-	-	-	-	-	-	-	-	-	-

Table S6: nDLS results after multipeak deconvolution for SPNPs series. A multipeak (LogNormal) deconvolution was utilized to extract the average sizes (d_1, d_2), distribution breadth (σ_1, σ_2), and population fraction (α_1, α_2) for both the individual particles (population 1) and the transient clusters (population 2). (In the main text, for all SPNPs, d_1 refers to the average for the smallest diameter distribution and d_2 refers to the average of any larger diameter distribution.)

SPNPs	Population 1			Population 2		
	Dia. (nm)	σ (nm)	α (%)	Dia. (nm)	σ (nm)	α (%)
**HEM**	91	15	17	347	119	83
**HEMHSA**	97	19	65	455	159	35
**TF**	125	29	29	233	98	71
**TFHSA**	80	16	83	326	136	17
**MUC**	39	8	95	180	93	5
**MUCHSA**	30	4	7	68	21	93
**INS**	35	6	92	144	70	8
**INSHSA**	64	15	79	138	65	21
**HSA**	46	10	16	222	121	84

Table S7: Physiochemical properties of proteins.

Protein	Molecular weight	Ratio of hydrophilic residues / total number of residues [12]	Isoelectric point	References
HSA	66.5 kda [1]	41%	4.7 [2]	[1,2]
Transferrin	79 kDa [3]	38%	5.6 [4]	[3,4]
Mucin	4000-5,500 kDa [5,6]	19%	2.75 [7]	[5,6,7]
Hemoglobin	64.5 kDa [8]	30% - alpha subunit<br>29% - beta subunit	7.0 [9]	[8,9]
Insulin	5.808 kDa [10]	29%	5.5 [11]	[10,11]

Table S8: SPNP jetting solution formulations.

Formulation	Protein (mg)	Crosslinker (mg)	Water (mL)	Ethanol (mL)	Acetic Acid 10% (mL)
HSA	50	5	400	50	
TF	50	5	400	50	
HEM	50	5	400	50	
INS	50	5	405	50	45
MUC	10	1	400	50	
TF/HSA	25/25	5	400	50	
HEM/HSA	25/25	5	400	50	
INS/HSA	25/25	5	427.7	50	22.5
MUC/HSA	10/10	2	430	50	

References

1. Elzoghby, A. O., Samy, W. M. & Elgindy, N. A. Protein-based nanocarriers as promising drug and gene delivery systems. *J. Control. Release* **161**, 38–49 (2012).
2. Joshi, M., Nagarsenkar, M. & Prabhakar, B. Albumin nanocarriers for pulmonary drug delivery: An attractive approach. *J. Drug Deliv. Sci. Technol.* **56**, (2020).
3. Gomme, P. T. & McCann, K. B. Transferrin: Structure, function and potential therapeutic actions. *Drug Discov. Today* **10**, 267–273 (2005).
4. HOVANESSIAN, A. G. & AWDEH, Z. L. Gel Isoelectric Focusing of Human-Serum Transferrin. *Eur. J. Biochem.* **68**, 333–338 (1976).
5. Fogg, F. J. J. *et al.* Characterization of pig colonic mucins. *Biochem. J.* **316**, 937–942 (1996).
6. Schömig, V. J. *et al.* An optimized purification process for porcine gastric mucin with preservation of its native functional properties. *RSC Adv.* **6**, 44932–44943 (2016).
7. Curnutt, A., Smith, K., Darrow, E. & Walters, K. B. Chemical and Microstructural Characterization of pH and [Ca2+] Dependent Sol-Gel Transitions in Mucin Biopolymer. *Sci. Rep.* **10**, 21–23 (2020).
8. Zhao, Y. *et al.* A novel nanoparticle drug delivery system based on PEGylated hemoglobin for cancer therapy. *Drug Deliv.* **26**, 717–723 (2019).
9. Devineau, S. *et al.* Change of the isoelectric point of hemoglobin at the air/water interface probed by the orientational flip-flop of water molecules. *Phys. Chem. Chem. Phys.* **19**, 10292–10300 (2017).
10. Mo, R., Jiang, T., Di, J., Tai, W. & Gu, Z. Emerging micro- and nanotechnology based synthetic approaches for insulin delivery. *Chem. Soc. Rev.* **43**, 3595–3629 (2014).
11. Kivlehan, F., Lanyon, Y. H. & Arrigan, D. W. M. Electrochemical study of insulin at the polarized liquid-liquid interface. *Langmuir* **24**, 9876–9882 (2008).
12. Bachem.com. 2020. *Bachem - Peptide Calculator*. [online] Available at: <https://www.bachem.com/service-support/peptide-calculator/> [Accessed 25 December 2020].
